# Relapses in Diphtheria

**Published:** 1907-11-30

**Authors:** 


### RELAPSES IN DIPHTHERIA.

The various aspects of a disease so common and so important as diphtheria must of necessity interest every member of the profession. It is not only the rare and the obscure that demand investigation and attention, and medicine owes its vastest tribute of recognition to those who have investigated, or who are investigating, common ailments and their sequelæ. Diphtheria, as a disease, demands the closest and most careful investigation, and in the tabulation of results and statistics, which in the end will do much to throw light on the obscurities of its protean manifestations, the general practitioner can afford much help and be of great assistance to the specialist or the ultimate compiler. It is true fever hospitals, where large numbers of cases are seen yearly, and where every opportunity for research is at hand, offer special facilities for the study of the disease, but there are some aspects of diphtheria which can be but imperfectly studied at the fever hospitals. One of these aspects, and in many ways one of the most interesting, is the occurrence of relapses.

That relapses are by no means rare in diphtheria has been admitted from the earliest times by various authors. Even before the days when the disease, as a clinical entity, was universally recognised, it was recorded, of cases which in modern times would have been unhesitatingly labelled as diphtheria, that "exacerbations and recurrences" took place. Later on, when the distinction of the disease from other affections of somewhat analogous clinical nature was recognised, equally striking evidences of recurrences and relapses were placed on record by various observers. English writers, among them Morell Mackenzie and Downes, laid stress on the severity of "second attacks of diphtheria," but the fact that relapses were more severe than the primary attacks was not generally admitted, and Continental observers, indeed, regarded second and third attacks as of relatively less severity than a primary attack.

The latest contribution to the study of relapses in diphtheria is an able paper, by Dr. J. D. Rolleston, of the Grove Fever Hospital, published in a recent number of the *British Journal of Children's Diseases*. From an unusually rich experience of such secondary attacks, Dr. Rolleston draws certain conclusions which, although we do not expect that they will be accepted in their entirety, cannot but be of great interest to practitioners. Summarised his conclusions are as follows: That relapses occur in a little more than one per cent. of all cases; that they are less frequent than late tonsillitis in convalescence from diphtheria; that they do not occur before the third week; that they are usually milder than primary attacks; and that their causation is obscure. Even more important, from a practical point of view, are his conclusions on the subject of differential diagnosis and of treatment. Usually, he remarks, the diagnosis of a relapse is easy, but it is imperative to distinguish it from the condition known as pseudo-relapse, for which Dr. Rolleston has suggested the name "Angina redux." This is a late serum phenomenon, usually appearing a fortnight after the first injection, and characterised by a circinate erythema, pyrexia, adenitis, arthritic pain, and an enanthematous deposit on the tonsils. An even more important condition demanding differential recogn[illegible]tion is scarlet fever, in which the sore throat appe[illegible] before the rash, although it is unusual to fi[illegible] definite patches on the tonsils before the rash is well developed. A third condition is late tonsillitis, in which the discrete nature of the deposit and the absence of the Klebs-Loeffler bacillus are distinctive. As treatment Dr. Rolleston recommends anti-diphtheritic serum in small doses, not exceeding 8,000 units. In adults he regards it as safer to delay or withhold antitoxin altogether, and to trust purely to local treatment. All the cases of relapsing diphtheria yield readily to small doses of antitoxin, and the prognosis is usually favourable, both as regards the acute attack and the absence of subsequent complications.

